# Health professionals’ initial experiences and perceptions of the acceptability of a whole-hospital, pro-active electronic paediatric early warning system (the DETECT study): a qualitative interview study

**DOI:** 10.1186/s12887-022-03411-1

**Published:** 2022-06-24

**Authors:** Bernie Carter, Holly Saron, Sarah Siner, Jennifer Preston, Matthew Peak, Fulya Mehta, Steven Lane, Caroline Lambert, Dawn Jones, Hannah Hughes, Jane Harris, Leah Evans, Sarah Dee, Chin-Kien Eyton-Chong, Gerri Sefton, Enitan D. Carrol

**Affiliations:** 1 Edge Hill University, Ormskirk, UK; 2 Alder Hey Children’s NHS Foundation Trust, Liverpool, UK; 3 University of Liverpool, Liverpool, UK; 4 Liverpool John Moores University, Liverpool, UK

**Keywords:** Acceptability, Deterioration, Escalation of care, Implementation, Paediatric early warning score, PEWS, Utility

## Abstract

**Background:**

Paediatric early warning systems (PEWS) alert health professionals to signs of a child’s deterioration with the intention of triggering an urgent review and escalating care. They can reduce unplanned critical care transfer, cardiac arrest, and death. Electronic systems may be superior to paper-based systems. The objective of the study was to critically explore the initial experiences and perceptions of health professionals about the acceptability of DETECT e-PEWS, and what factors influence its acceptability.

**Methods:**

A descriptive qualitative study (part of The DETECT study) was undertaken February 2020–2021. Single, semi-structured telephone interviews were used. The setting was a tertiary children’s hospital, UK. The participants were health professionals working in study setting and using DETECT e-PEWS. Sampling was undertaken using a mix of convenience and snowballing techniques. Participants represented two user-groups: ‘documenting vital signs’ (D-VS) and ‘responding to vital signs’ (R-VS). Perceptions of clinical utility and acceptability of DETECT e-PEWS were derived from thematic analysis of transcripts.

**Results:**

Fourteen HPs (12 nurses, 2 doctors) participated; seven in D-VS and seven in the R-VS group. Three main themes were identified: complying with DETECT e-PEWS, circumventing DETECT e-PEWS, and disregarding DETECT e-PEWS. Overall clinical utility and acceptability were deemed good for HPs in the D-VS group but there was diversity in perception in the R-VS group (nurses found it more acceptable than doctors). Compliance was better in the D-VS group where use of DETECT e-PEWS was mandated and used more consistently. Some health professionals circumvented DETECT e-PEWS and fell back into old habits. Doctors (R-VS) did not consistently engage with DETECT e-PEWS, which reduced the acceptability of the system, even in those who thought the system brought benefits.

**Conclusions:**

Speed and accuracy of real-time data, automation of triggering alerts and improved situational awareness were key factors that contributed to the acceptability of DETECT e-PEWS. Mandating use of both recording and responding aspects of DETECT e-PEWS is needed to ensure full implementation.

**Supplementary Information:**

The online version contains supplementary material available at 10.1186/s12887-022-03411-1.

## Background

Paediatric early warning systems (PEWS) [1–3] and PEW scores [4–6] are based on a child’s vital signs and other factors such as parental concern [7, 8]. They alert health professionals (HPs) to signs of a child’s deterioration, with the intention of triggering an urgent review and escalating care, as needed [9], and reducing emergency transfer to critical care, cardiac arrest and death [10–14]. Recording PEW scores can be paper-based or electronic [3, 9, 15]; e-scoring has advantages in comparison to paper-based scoring in terms of enhanced safety benefits including greater time efficiency, reduction in human error and instant availability of the recorded data [16, 17]. There is a drive for PEWS to be embedded in the care of children in hospital [13, 18], but in the UK their use is not consistent [3]. The evidence base for the effectiveness of PEWS and PEW scores is ambiguous [18, 19].

### Defining clinical utility and acceptability

We adopted a narrow definition of clinical utility; does the technology do what it is supposed to, and does it perform its designated function [20]. We chose a multifaceted definition of acceptability as proposed in the Theoretical Framework of Acceptability (TFA) [21] since we appreciated that implementation, adoption and assimilation of technology in healthcare systems is inherently complex [21–25]. The TFA is composed of seven component constructs: ‘affective attitude’, ‘burden’, ‘ethicality’, ‘intervention coherence’, ‘opportunity costs’, ‘perceived effectiveness’ and ‘self-efficacy’.

### The DETECT study

The Dynamic Electronic Tracking and Escalation to reduce Critical care Transfers (DETECT) study [17] implemented a proactive end-to-end deterioration solution (the DETECT surveillance system) across a tertiary children’s hospital with the aim of screening children for early signs of serious deterioration or sepsis and reducing complications and emergency transfers to critical care following deterioration in hospital. The DETECT surveillance system is supported by System C’s CareFlow Connect and Vitals (paediatric version) apps. These apps were modified for the study and are known as DETECT e-PEWS. DETECT e-PEWS is used by HPs to document vital signs on iPods (Supplement 1) and escalate concern (Supplement 2) and to respond to alerts of deterioration triggered by the system using iPods, iPads or by personal mobile device (Supplement 3). Alert thresholds for the study were set to signal children whose deterioration trajectory suggested potential transfer to the high dependency unit (scores 6–9) or paediatric intensive care unit (scores 10+).

Within the DETECT study we chose to measure both clinical utility and acceptability as this meant that, across both concepts, we were measuring a broad range of important factors. The main focus of the qualitative study, reported in this paper, was on the acceptability so we chose a multifaceted definition of acceptability as proposed in the Theoretical Framework of Acceptability (TFA) [21] since we appreciated that implementation, adoption and assimilation of technology in healthcare systems is inherently complex [21–25]. The TFA is the first robustly developed framework, that provides conceptually distinct constructs that reflect the key dimensions of acceptability. We selected the TFA as it offers a coherent evidence-based to defining and assessing acceptability in healthcare [21]. The TFA is composed of seven component constructs: ‘affective attitude’, ‘burden’, ‘ethicality’, ‘intervention coherence’, ‘opportunity costs’, ‘perceived effectiveness’ and ‘self-efficacy’. However, we were also interested in health professionals’ perceptions of clinical utility so we adopted a narrow definition of clinical utility; does the technology do what it is supposed to, and does it perform its designated function [20]; other parts of the larger study addressed wider and different aspects of clinical utility.

In this paper we present the findings of a qualitative, interview-based sub-study of HPs who were in the initial months of using the DETECT e-PEWS as part of the DETECT surveillance system.

## Methods

The aims and research question were: What are the experiences and perceptions of HPs about the acceptability (primary aim) and clinical utility (secondary aim) of DETECT e-PEWS and what factors influence acceptability?

### Study design

This descriptive qualitative design was selected as this allows the study to be informed by naturalistic principles, aims to generate a clear description of the phenomenon under study but does not aim to develop theory [26, 27]. The study was reviewed and approved by the North-West, Liverpool East Research Ethics Committee (IRAS ID: 215339). This study followed the Consolidated Criteria for Reporting Qualitative Research (COREQ) guideline.

### Participants and setting

HPs working at Alder Hey Children’s Hospital, a tertiary setting in the UK, were invited to participate in the interviews. Recruitment occurred between February 2020 and February 2021. Any HP who used DETECT e-PEWS was eligible to participate.

Convenience sampling was used to recruit participants either by expression of interest at the end of an associated DETECT study survey (paper in submission), by research nurses on the wards or snowballing by participants. Initial contact was by email with an information sheet; then a mutually convenient time was arranged for a telephone call where any questions about the study were answered, consent was gained, and the interview undertaken.

### Data collection

Single, semi-structured audio-recorded interviews were conducted by telephone (further details-Supplement 4). The interview schedule (15 questions) covered key demographic data, relevant experience with vital signs, raising a concern and/or responding to a child’s potential or actual deterioration and questions about the acceptability of DETECT e-PEWS (Supplement 5).

### Analysis

The interviews were analysed (BC, HS) using the five stages of thematic analysis [28]; familiarisation, generating initial codes, searching for themes, reviewing themes and producing report (details-Supplement 4).

## Results

Fourteen HPs participated (*n* = 12 nurses, *n* = 2 doctors); a further 25 expressed initial interest but failed to respond to texts/emails (after three invitations spaced over a few weeks with no reply, no further invitations were sent). Participants could be broadly categorised into two groups according to DETECT e-PEWS role:

- Documenting Vital Signs (D-VS) group: assessed and recorded vital signs (Assistant Nurse Practitioner (*n* = 1), Staff Nurses (*n* = 6)); and
- Responding to Vital Signs (R-VS) group: reviewed patients and responded to tasks and alerts (Nurse in Charge (*n* = 2), Advanced Nurse Practitioners (*n* = 3), and Doctors specialising in general paediatrics (n = 2)).

HPs noted that the implementation of DETECT e-PEWS was just one of a series of recent changes e.g., updates to electronic patient record (Meditech), a new bleep (paging) system and a new Acute Care (ACT) response team. Although implementation of the documentation component started 6 months pre-COVID-19 pandemic, implementation of the response component occurred close to the lockdowns and HPs had to accommodate changes required due to the COVID-pandemic.

**Fig. 1:**
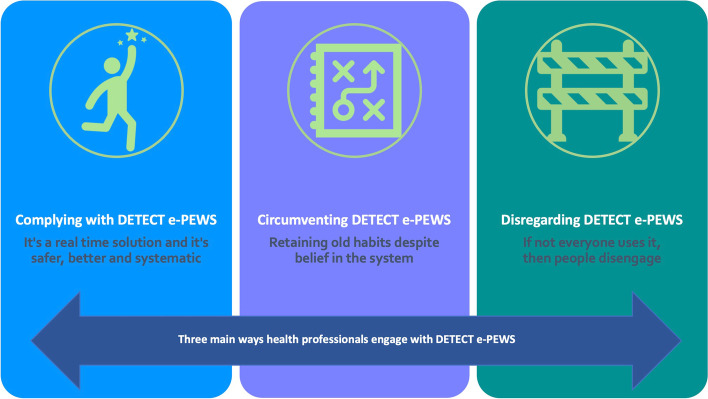
Three main themes reflecting the ways in which health professionals engage with DETECT e-PEWS

Three key themes and eight sub-themes were identified (Fig. 1): Complying with DETECT e-PEWS; Circumventing DETECT e-PEWS; and Disregarding DETECT e-PEWS. Detailed quotes appear in Table 1. These themes relate to the clinical utility and acceptability of DETECT e-PEWS and reflect how HPs responded to and engaged with the technology.

**Table 1:** Overview of themes and sub-themes with supporting quotations (by group)

**Theme 1: Complying with DETECT e-PEWS**	
**Documenting Vital Signs Group (D-VS)**	**Reviewing and Responding to Vital Signs Group (R-VS)**
**Initial impressions**	
I was trained in a hospital where we didn’t have that so I just thought it was quick and easy to carry it in, put it in your pocket, go into the room and you can just do everything in real time rather than writing it on a piece of paper and going out and sometimes you can get distracted … …. I thought that was really effective device to have (D-VS4). I think I thought that it was quite systematic the way it goes through then different sections of the onset that you have to put in. I just remember thinking that it was a big change and that we were never going to use it and that isn’t true as we do use it just not all the time (D-VS5). I’m confident in the numbers I’m putting in … at first I was thinking, ‘If I put, like, a wrong digit in, it’s going to start sending … all sorts of alerts and they’ll think the child’s got desperate,’ but it’s worked out absolutely fine (D-VS2).	It takes a little while to get your head round and knowing where everything is and knowing what you need to do …. [but] I don’t find them confusing anymore …. and I think also the more and more we have used it the more we appreciate the advantages of it … …. It’s a lot simpler information wise, [there’s] a bit less messing [finding an available computer] … vital [signs] are getting noted straight away (R-VS2). I liked it, from the beginning (R-VS4). It’s all just there in front of you. And you can pick it up as and when you need it (R-VS5).
**Clinical utility issues**	
If everything was on the same device [for entering], so if all the fluids and everything like that were also on there, then I would find it a lot better (D-VS3). Only the odd time I think I’ve put obs on and they’ve not registered as being done but then I’ve spoke to people in DETECT and got that looked at. It’s not a common thing (D-VS6). I think maybe once or twice I’ve started on my patient and then I’ve released oh god, that’s not my patient … … you’ve got to double-check (D-VS1). Sometimes when you have got gloves on you press a number but it gives, say for instance a couple of times I have wanted to put in 24 resps and gone to 4 but the 4 hasn’t linked up (D-VS4). A couple of times we’ve put in observations and they’ve actually not gone through to Meditech. There’s like a couple of hours missing and stuff and sometimes like if the nurse in charge has spotted that before you, they’ll come over and say ‘Have you done obs for the last such and such hours’ and I’m like ‘Well yes, I have actually’ (D-VS3). On our ward … it’s fast turnover, so …. if [new admission is] not booked in on the system, you can’t actually get their name up on the device -- so sometimes you have got to wait … (D-VS2). To be honest, I thought it was extra work … because we have bedside computers in every bed space so for us, it was like having to use an extra device because our PEWS were on Meditech to begin with so we’re logging on every hour to put fluids on there at the moment and before. So it felt like I had to access another device. … ..I still don’t think it’s right for the HDU environment to be honest … … .because we’re accessing the computers to do the fluid anyway so it was easy enough to just put our PEWS in on there during that time while we were putting the fluids in (D-VS3).	[Problems] have been resolved quickly (R-VS1). Without the DETECT device our job would be harder then, you know, as simple as it is (R-VS5). I’ll often already have one or two baton bleeps and then you can be caught holding an on-call bleep as well … … so I’d say two to three devices will often be the case, so it does add another one (R-VS7). I guess the main thing is logging in. If you forget your password, usually that has got to be the main one (R-VS2). The internet signal [can be a problem] …. If we’re in the stairwell, for instance …. and then we won’t get an alert … and then it takes a while to kick in. That’s unfortunately the build of the hospital not the device (R-VS5). Our ward manager has very strongly started pushing it and making it very much priority, we use it for vitals, notifying doctors when we are concerned about patients and things like that … (R-VS2). Our ward manager was very good at just making sure going round saying can we please charge the devices please put them back on charge. We have had a couple of staff who have accidentally taken it home in their pocket and things like that because you know you think is this my phone, a calculator just routinely, I think [manager] put signs on the doors saying ‘Is it in your pocket’? (R-VS2). It depends on whether those alerts are actually coming to your device, whether they’re pinging or not and whether anybody’s actually looking at it (R-VS6). I think that idea of a recurrent [alert] alarm could support [DETECT] … a couple of times …. I’ve not heard alarm [in busy area that’s noisy], and because it hasn’t gone off again, I’ve missed it (R-VS4).
**It’s systematic, real-time, and it’s got my back**	
With a paper towel you record your obs and no one was seeing that were they? Only you (D-VS1). If you’re walking round with bits of paper, you can always lose them, or, like, forget to put them on the system, but if they’re recorded straight away on [DETECT], anyone can see them, and they’re done in, like, real time (D-VS2). Recording obs in real time and that gives a good time line if a child does deteriorate, because then you can say I did an obs at this time, I was in the room and right there and then rather than I did the obs and then 10 minutes later I documented them and counted up what the score was and contacted the doctor, electronically it’s all done right then and there for you it does scoring (D-VS4). Being newly qualified and anxious in my job because I am new and you have to build confidence it is good for me to know that I’ve got that as a backup if I am concerned about a patient as I can just click that button and I have done it in the past and there is a phone call straight to the pod and there is a doctor saying I have just had an alert for patient such as such and then they can come and review the patient for you. (D-VS4). I think the neurological assessment is quite good when you are putting the GCS in as some people don’t remember to do all the steps or know exactly where to place or if you take the device in with you, you can go through it in the room with the patient and it’s also got the sizes of the pupils it doesn’t just say like 2, 3, 4 it’s the size that you can compare it to as well, so that’s quite useful (D-VS5). I just thought ‘Oh another device and an extra bit of work’, but it actually isn’t, it’s faster (D-VS1). At first, when I was first putting obs on, it took me for ever, ‘cause I was, like, submit, and then making sure the numbers was right on there, but obviously because I’m used to it now and I know what questions are coming next, I’m quite quick on them, so I know exactly what I’m inputting and, you know (D-VS2). I think it is good for inputting, like, observations at real time, so if a patient does react, you can then communicate at the right time, and quickly enough, to get in touch with doctors and things like that, to obviously observe the patient and, you know, review them and things (D-VS2). Yeah, I think it saves on paper as well, having loads of paper and trying to rifle through paper as a student trying to find the relevant stuff that you need even with notes and stuff or obs charts, but here you just do it on the DETECT …. look at the vital signs, get the graph up to look at how their obs have changed in the last 24 hours or last 12 hours … … … you can just find exactly what you want, when you want just through technology (D-VS4).	I suppose you do have to really concentrate on the observations, and you have to read what you writing in not just like, and people don’t do it wrong in that sort of sense, people don’t skip past things because they can’t, because you can’t skip past it, you have to do it. And so you can’t just say, oh, we won’t take the temperature this time, or will just work through the rest time. Because you’re having to write down you’ve not done it so that is true actually the results were getting actually are better (R-VS5). I think in terms of data input it’s made a massive difference …. I do think because the data entry is quicker, I think information is considered more quickly (R-VS7). You can access it from anywhere and … see [data] at any point of time and … the notes you used to write on paper used to get lost (R-VS6). So I was straight in with the patient then and with the doctor. And we were able to bring the child who needed intubating on the ward …… but it was a controlled step up to critical care … …. we were there at the time they needed us (R-VS5). It helps us to get there [to child] quicker, and it does what it says on the tin, really, truthfully (R-VS4). I can see improved communication in the hospital … ..where a high PEW has been triggered, I have been alerted and as soon as I have gone a couple minutes later someone from the ACT team has come as well so that’s actually quite reassuring for the nurses as well (R-VS3). It ties everything together - before everything was very separate - …. It’s pulling it into one place. It’s all accurate and [real] time and all that so all that has benefitted us quite a lot. Now [we do handover using the devices] at the bedside … .. it’s very much easier you don’t have to get access to sit down at a computer. Way quicker, way quicker (R-VS2). I just thought they were picked up quickly and the few times when I have been concerned the obs have already been on to it so to me that’s working isn’t it? (R-VS1). I like the idea of them being able to pin tasks and you being able to reply to the task rather than getting bleeped and having to interrupt what you’re doing and not knowing whether your bleep is not that urgent (R-VS7). I think sometimes, for the medical teams because I work closely with them, the idea of generating a task enables them to prioritise their workload doesn’t it? If you need to prescribe some paracetamol for somebody and you need to do a cannula then you know they can prioritise what they need to do where I think doctors are probably getting bleeped a lot more than what they need to be now for more simple things (R-VS3).
**It improves situational awareness**	
I mean whenever I’ve been in charge it’s really good because you get an automatic alert if a patient scores a high PEW or there’s a sepsis concern so you’re automatically made aware of it … and I can escalate any concerns or contact the medical team and we’re using it for electronic handover (D-VS6). Quick because if a child’s PEW is a certain amount on the ward the nurse in charge will come straight to you and say I’ve noticed that such a such is 4 why is this? What are your reasons, how are you feeling? (D-VS4).	I jump onto my DETECT thing and I can pull up the PEWs chart and I like the fact that it does it in an old fashioned chart so you can see trends a little bit easier [than Meditech] and also I like to be able to see what the nurse’s handover is as well on it (R-VS3). Our [ACT] team are able to tag patients that have stepped out of critical care, or acute admissions, and children within 24 hours that come in particularly poorly … so we’ve got a vast view on which patients we need [to review] (R-VS5). Definitely quicker, we [ACT] pick up alerts and contact the ward straight away.... Before [DETECT] we wouldn’t even know that these kids were PEWing on the wards at all until we walked around the ward (R-VS5). I think we would rather have a PEW come up and know that its normal for them than not have something come up and miss something completely as that would just be tragedy (R-VS2). We can see if someone’s [child’s] struggling a little bit.. shows on their PEW, and we can … contact the ward earlier to try and help out (R-VS4). Yeah, that [nurse concern and parent concern option] really makes a massive difference, I think. Because obviously there’s some kids [underlying condition].. that will always trigger the PEW. But that’s just them; they’re fine like that. You know, there’s nothing—there’s no intervention to be done; nothing needs to happen. So we can ring and say, ‘What’s the matter? Something’s wrong. Is there anything we can help you with? D’you need us to do anything?’ (R-VS4). I think that it’s created a culture of everyone taking [deterioration] very seriously, not that everyone didn’t take [deterioration] seriously before, everyone’s always taken things seriously, but it’s now a priority to be using [DETECT] and you know engaging with it …. actively … [it’s] putting information to the forefront (R-VS7). I use the Careflow App on my phone … every day that I’m in hospital and some days I’m not in hospital to keep tabs (R-VS7). I do feel like they have improved communication … especially handover when they are updating properly … you know what needs to be done next …. it really helps when the doctor comes on the ward as they have got all the vitals already before they even step on the ward. So, yeah, communication wise it definitely has improved (R-VS2).
**It can create distance from and support closeness with patients**	
I think it can limit conversations with parents - they might not want to speak to you … if they see you on a device … ‘cause they might think they’re distracting you (D-VS2). You could be in a room with a child with a real high score and it is not safe to leave them and you have to be there monitoring them. So on the device it allows you to say are you concerned, yes, are the parents concerned, yes or no, and then do you want to contact someone and you can click yes and that goes through to either a doctor or links up to the nurse in charge so you are able to stay with your patient but also to alert the staff with your concerns without leaving the room which I think is a really good idea (D-VS4). If you’ve got a baby on CPAP who’s dead agitated for instance … you can [soothe the baby] and do your obs … you don’t have to leave your patient to come away and either go on the computer or like it used to be on paperwork (D-VS1).	[DETECT] is quick, and it gives us real time data and all that kind of stuff. But actually, the process of it feels distant from your nursing care (R-VS5). I think sometimes your concentration is pulled more towards your device rather than the child (R-VS5).
**It accommodates clinical judgement**	
To be honest again the PEWs we do obviously follow the PEWs system on our ward but it’s a lot different to on the ward because our PEWs are basically anything over 6 because a lot of ours that have a PEW of 4 anyway just because of their oxygen levels and things like that. So we’ve moved ours up to 6 so I yeah, we just kind of know the difference in numbers instead of going with PEWs because a lot of ours will sit at 75 like one SATS above 75 anyway so that’s going to alert as a PEW there because, but that’s normal for that patient. They’ll normally sit in two litres of oxygen but again they’ll PEW one or two for that because they’re needing oxygen but yeah, that’s their normal anyway. So just a lot of these things like so we’ll go up to our nurse in charge and say look, I’ve just PEWed a 6 for this patient but they normally PEW a 4 anyway so they’re actually not, it’s not extreme that we need to ring a consultant or whatever or ring somebody, ring a doctor (D-VS3).	They were getting alerts from the DETECT devices and saying like, this child is poorly and this child was poorly, but they know that child and they know that that is normal for that child, so actually what was happening was they were getting bleeped so many times and it was like they were filtering through it all … … whereas when the nurses actually bleep you, not the DETECT devices bleep you, I think it was more accurate that way and I think there is always those patients that have a high PEW score and when you log in, if you know them you can ignore it, if you don’t know them you think, ‘Oh, is that a problem?’ so I think actually as a way of highlighting the people with the worst obs as sick, I haven’t found it useful (R-VS7). The ward we are on is fantastic there and obviously a lot of the time we will say to the doctors, you know, patient X has PEWed this but obviously we are not having any nursing concerns but we have to tell you (R-VS2).
**Theme 2: Circumventing DETECT e-PEWS**	
**It’s easy to fall back to old ways**	
I use it … Every shift probably multiple times a shift … … I don’t use the computer for anything …. [but] I probably use them at the bedside less than 50% of the time … I still tend to do obs as I used to, write them down on a bit of paper and then fill them in when I come out of the cubicle. It’s just a habit really that I haven’t really changed. … … …… I tend to do it straight away as soon as I come out, but say if I am really busy and have got other things to do, then it might be like 10 minutes and I will do my whole set of 4 obs first and then put them all in on the system (D-VS5).	I carry mine all the time [but] the girls on the ward don’t generate tasks specific for me … .because I am ward based and I am walking around the ward all the time, so if there is an issue they will just grab me rather than generate a task (R-VS3).
**It’s not reliable in terms of getting a response,**	
If it was something that they have PEWed high then I do feel more comfortable just bleeping them and speaking to the over the phone. Yeah so you know they have definitely read it and are definitely aware of it, you have heard them talk to you (D-VS5).	I think people have been nervous and they haven’t been reassured that it is going to get picked up and they don’t want to leave there patients but they start to get a bit paranoid as a nurse as you start to think I need this patient to be seen (R-VS2). Because a lot of the medics haven’t been using the task list properly, they’ll see a task but the medics wont complete the task or respond to the task, so the wards will then bleep and they just got out the habit of it, it wasn’t really working because everyone wasn’t on board with it hopefully that’s getting sorted with plans ahead for that (R-VS5). I think [nurses] probably because their experience I expect has been that when they put a job on Careflow no one does it and they have to bleep them anyway. I think they disengage (R-VS7).
**Theme 3: Disregarding DETECT e-PEWS**	
At the start we were trying to use the way to alert doctors but now we’ve got our own consultants on the ward so we don’t tend to use, we don’t use the detects for that any more. We just go to our own doctors (D-VS3). Not too sure if they [doctors] take [DETECT] as serious as we [staff nurses] do because they don’t use it as much or they’re not as reliable with it and don’t count on it as much as we do (D-VS6).	The doctors I mean, especially the surgeons, definitely the surgeons don’t use them. The surgical wards definitely don’t bother for that reason. Medical ones were better but I think it’s definitely sort of tailed off really (R-VS5). I would say another thing that we noticed and struggled with initially was when we put through concerns to the doctors a lot of the time they weren’t getting picked up on the other end so we would have to go on and bleep and do all the things we were doing so it just doubled up on the workload basically (R-VS2). Alert fatigue (R-VS6). I think people have been nervous and they haven’t been reassured that it is going to get picked up and they don’t want to leave there patients but they start to get a bit paranoid as a nurse as you start to think I need this patient to be seen (R-VS2). It’s not massively used at the moment it’s gone a bit by the wayside but we are trying to reintroduce the task and going to give it another push it a bit more hopefully (R-VS5). I think when they introduced it, they introduced, apart from the bleeps, we had to carry other device, I think that was impractical because then you were carrying your mobile, your bleep, and a third device, I mean, you don’t have so many pockets (R-VS6). So ultimately, the doctors not using them is then leading to other people not using them (R-VS5).

### Theme 1: complying with DETECT e-PEWS

Overall, this theme addresses the ways in which HPs positively engaged with DETECT e-PEWS, despite some challenges to utility, and how they complied with the intervention and followed the processes appropriately. However, it also shows that even those who complied and engaged experienced some tensions within the use of DETECT e-PEWS.

#### Initial impressions

Initial impressions of DETECT e-PEWS were generally positive and this supported compliance. Nurses (D-VS and R-VS groups) perceived it to be “systematic” (D-VS5), “quick and easy to carry” (D-VS4), and “real-time” (D-VS2). Some HPs (D-VS and R-VS groups) found using DETECT e-PEWS a “big change” (D-VS5) and initially “confusing” (R-VS2), although this was overcome as they gained confidence. One HP noted “the more we have used it the more we appreciate the advantages” (R-VS2).

#### Key issues related to clinical utility

There was agreement across both groups that the technology did what it was expected to and could perform its designated function. One HP explained **“**without the DETECT device our job would be harder” (R-VS-5). However, some aspects challenged its utility. In the D-VS group, the main complaint was that although vital signs and other data (e.g., blood sugar) could be recorded onto DETECT e-PEWS, fluid intake and output had to be inputted separately onto Meditech via a computer. The main objection within the R-VS group, and more of an issue for the doctors, was the desire to just have one device to carry as some were already carrying “one or two baton bleeps [pagers] and …. an on-call bleep as well” (R-VS7).

Some problems challenged utility in the early weeks of implementation, e.g., user errors such as forgetting the password, or entering data for wrong patient, and interface errors such as vital signs not being saved to Meditech. Other issues included poor internet signal, not hearing the alert alarm in noisy environments, and non-recurrent alert alarm. In settings where each bed had a computer the value of DETECT e-PEWS seemed less convincing as it was “like having to use an extra device” (D-VS3). Problems, where they occurred, were said to be “resolved quickly” (R-VS1).

#### It’s systematic, real-time, faster and it’s got my back

The aspects of DETECT e-PEWS that were seen to be the most positive and contributed to acceptability were those related to speed, “it’s faster” (D-VS1), accuracy of data input, real-time availability of vital sign data, triggering of alerts, quality of the graphs and ability to easily review patients; “it’s all just there in front of you. And you can pick it up as and when you need it” (R-VS5).

The D-VS group often referred to the benefits of DETECT e-PEWS over the previous system which involved inputting delays due to having to find an available computer. Via DETECT e-PEWS they “recorded straight away … anyone can see them … in real time” (D-VS2).

The immediacy of real-time data entry was beneficial for the R-VS group who stated that DETECT e-PEWS meant that information was “considered more quickly” (R-VS7), it had improved bedside handovers, improved the speed of response as it “helps us to get there [to child] quicker” (R-VS4) and had resulted in improved care of deteriorating children, “it was a controlled step up to critical care … we were there at the time they needed us” (R-VS5).

DETECT e-PEWS helped build confidence in newly qualified nursing staff, and the automated scoring was positively evaluated. All HPs in the D-VS group talked about the system acting as a back-up as it created an audit trail of their actions (e.g., documenting vital signs, creating tasks, sending messages, escalating concern) as it “gives a good timeline if a child does deteriorate, because then you can say I did an obs at this time … I’ve got that as a backup” (D-VS4).

HPs perceived that data completeness was improved, as DETECT e-PEWS used a systematic approach and ensured HPs “don’t skip past things because they can’t” (R-VS4) and the charts were easy to access and review. The data could also be “access[ed].. from anywhere” (R-VS6).

An aspect liked by some of the R-VS group related to managing workload via the use of ‘tasks’ (e.g. requesting cannulation) which, when used effectively, reduced the number of times doctors were getting bleeped as they could “pin tasks and … reply to the task rather than getting bleeped” (R-VS7).

#### It improves situational awareness

A key reason for the acceptability of DETECT e-PEWS was improved situational awareness about serious deterioration and it was thought to have “created a culture of everyone taking [deterioration] very seriously …. [it’s] putting information to the forefront” (R-VS7).

Alerts triggered by a high PEW score or sepsis concern meant the HP at the bedside was “automatically made aware … and... can escalate any concerns” (D-VS6). HPs in the R-VS group were aware of such concerns and, through tagging patients vulnerable to deterioration, HPs had “a vast view on which patients we need [to review]” (R-VS5).

#### It can create distance from and support closeness with patients

An aspect that was commented on mostly by the D-VS group was that an alert could be triggered, or a concern raised at the bedside. This was seen as particularly important with a deteriorating child as “you are able to stay with your patient but also to alert the staff with your concerns without leaving the room” (D-VS4).

However, two HPs mentioned that they felt that using the iPod sometimes impacted on the social relationship aspect of nursing care either because parents “might not want to speak to [and disturb] you” (D-VS2) whilst entering the data or because the remote nature of reviewing patients meant that you felt “distant from your nursing care” (R-VS5).

#### It accommodates clinical judgement

DETECT e-PEWS had standardised algorithms underpinning the alert system which meant that alerts would trigger at a given threshold. This sometimes occurred in a patient who had a deterioration management plan in place and was no worse than previously. Staff could annotate alerts (6 and above, reflecting their real-time clinical judgement: “a lot of our [patients] that have a PEW of 4 anyway just because of their oxygen levels and things like that [so a 6 is not so high]” (D-VS3). Some HPs (R-VS group) suggested a high number of alerts about children with underlying health conditions could cause alert fatigue.

### Theme 2: circumventing DETECT e-PEWS

This theme addresses the ways in which HPs circumvented the processes associated with DETECT e-PEWS.

#### It’s easy to fall back to old ways

Even HPs who were positive advocates for DETECT e-PEWS sometimes circumvented the system; this reduced the system’s effectiveness and could reintroduce error. Typically, HPs (DV-S) entered data onto the iPod at the bedside, but some reverted to the old habits; in one instance this appeared to be routine: “I probably use them at the bedside less than 50% of the time … I still tend to …. write them down on a bit of paper and then fill them in when I come out of the cubicle” (D-VS5). In settings where a member of the R-VS group was readily accessible, staff subverted the task messaging aspect of the system as they made direct contact with the clinician and “just grab me rather than generate a task” (R-VS3).

#### It’s not reliable in terms of getting a response, we still need to bleep

An issue with the implementation was that whilst it was mandatory for vital signs to be documented via DETECT e-PEWS, it was not mandatory for responders (R-VS) to engage with the system. This meant that HPs (R-VS) did not always log in to the system so ward staff raising tasks were not always confident that they would get a response to tasks or alerts as “the medics … just got out of the habit of [responding to task]” (R-VS5). Thus, even committed HPs could be “nervous …. a bit paranoid” (R-VS6) if they were unsure a task had been picked up or felt they had waited too long for a response and would bleep a doctor anyway. Some felt more comfortable with the bleep system as it meant speaking to someone rather than just messaging as “you have heard them talk to you” (D-VS5).

### Theme 3: disregarding DETECT e-PEWS

Although nurses within the R-VS group engaged and valued the system, there were reports that many doctors in the hospital disregarded DETECT e-PEWS and did not commit or engage with the system. This meant that the review and response component of DETECT e-PEWS failed to consistently work effectively throughout the hospital as “the doctors not using them is then leading to other people not using them” (R-VS5). A typical response from the D-VS group was uncertainty whether doctors “take [DETECT] as serious as we [staff nurses] do because they …. don’t count on it as much as we do” (D-VS6). There was also a sense that some of the senior clinicians lacked commitment to the system and that this prejudiced the perceptions of the more junior staff, including any new starters; “if the people teaching [more junior doctors] don’t use it, the juniors are not going to use it” (R-VS5). When senior clinicians either resisted or stopped engaging, the result was that “everyone’s [doctors] just gone, “Oh, well there’s no point using them now” (R-VS4).

In addition to any speciality resistance (e.g., medical doctors perceived as being better at engaging than surgical doctors) and DETECT e-PEWS not being mandatory for doctors, its acceptability in the R-VS group was probably influenced since the response component was introduced around the time of the first lockdown from the COVID-19 pandemic. Problems related to “a lot of information overload [from other changes] (R-VS6) may also played a part. However, a further “push” (R-VS5) was planned in the future.

## Discussion

**Fig. 2:**
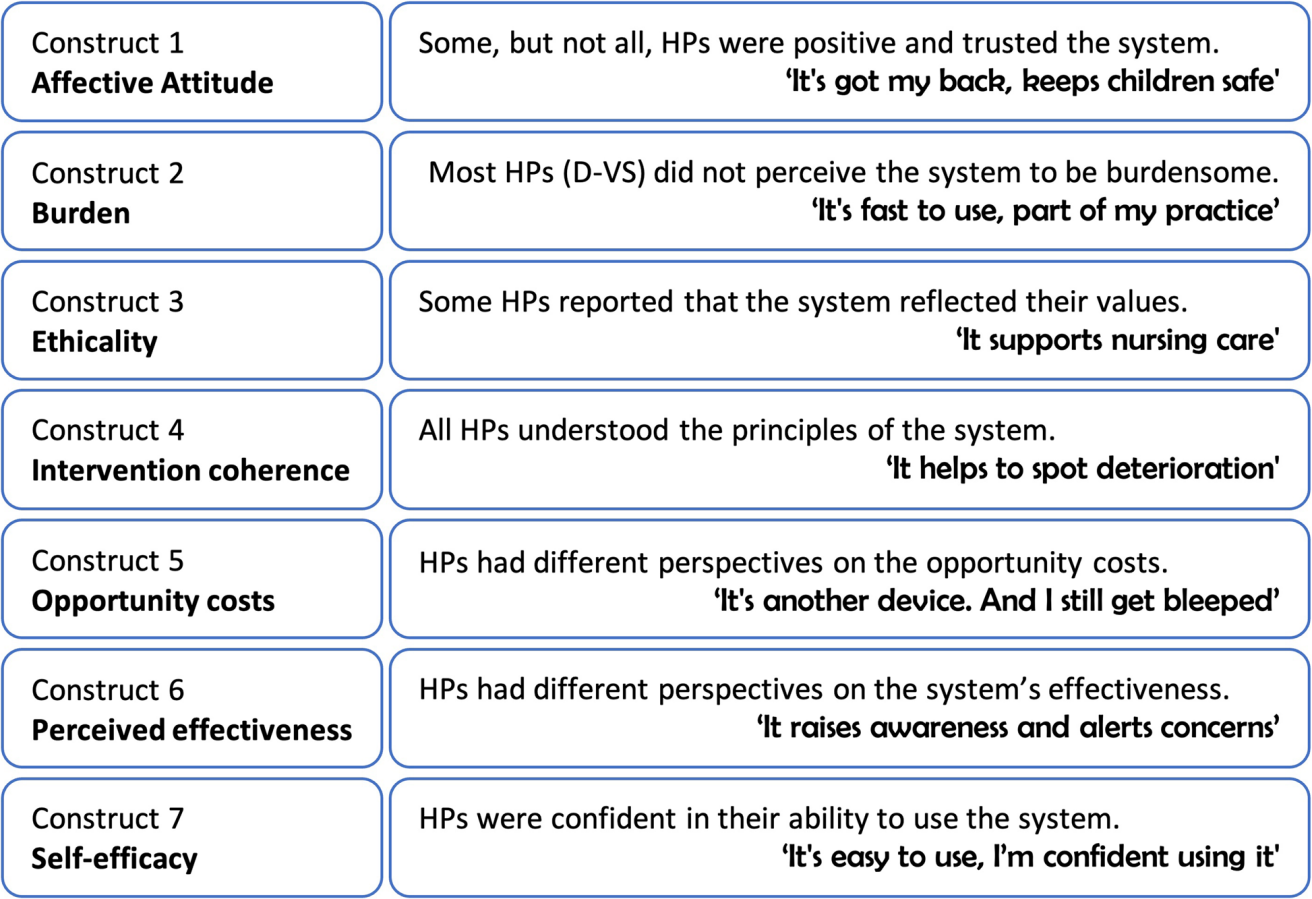
Domains of the Theoretical Framework of Acceptability [21] as applied to findings

The study aimed to explore the initial experiences and perceptions of HPs about the acceptability and the clinical utility of DETECT e-PEWS and what factors influence acceptability. The discussion draws on the seven constructs of the TFA to explore acceptability (see Fig. 2). The DETECT system aimed to resolve the key barriers that impact on identifying the deteriorating child, including incomplete documentation [29], data input not being in real time [30], incorrect calculation of scores [31], lack of/over-confidence [32], poor communication and/or delayed/non response to alerts [33, 34].

Overall HPs had positive ‘affective attitudes’ towards DETECT e-PEWS, welcomed its introduction and most overcame the initial problems they experienced. Nurses (D-VS and R-VS) had the most positive initial, and ongoing affective attitudes, whereas resistance by doctors to the use of DETECT e-PEWS was reported by all HPs; similar resistance is evident in other studies [16, 35]. Nurses’ positivity about DETECT e-PEWS reflected attributes reported in other studies including PEWS are systematic [36], reduce errors [9, 35], save time [9] and provide a safety net [32] via an audit trail. It demonstrated good clinical utility (it did what it was supposed to) with those who used it regularly.

The perception of ‘burden’ depended on the HPs’ level of engagement with DETECT e-PEWS and how much it benefited them in daily practice. The initial effort required to learn to navigate and use the system was quickly overcome for HPs in the D-VS group; the benefits accrued were clear cut and real time documentation was faster and not burdensome as seen in other studies of adopting PEWS [35]. However, the burden of carrying an extra device (R-VS group) was perceived as problematic by the doctors although not by the nurses.

‘Ethicality’ was evident. DETECT e-PEWS supported HPs values in relation to generating real-time information that could be easily shared, could reduce the risk of children deteriorating, and aligned with other values (e.g., accommodated clinical judgement, ability to stay at a sick child’s bedside). Implementation of change is enhanced when it fits HPs values) [37].

All HPs understood the purpose of DETECT e-PEWS and how it worked (‘intervention coherence’). HPs talked about how an effective whole system approach [38] such as DETECT e-PEWS can improve situation awareness (SA) (through alerts, raising concern, response to concerns) on both an individual level and at an organisational level (a culture of “everyone taking deterioration more seriously’), as seen in other studies [39, 40]. However, even confident HPs who expressed a clear understanding of benefits for children sometimes circumvented the system, deliberately choosing to revert to ‘paper towel’ (e.g., writing observations on paper towels and later transcribing into the device) techniques; other studies reveal adoption can be inhibited by persistence of paper-based approaches [34, 41].

Doctors were resistant to change, as reported in other studies [35]. The ‘opportunity costs’ were most evident for the doctors (D-VS group) who talked of having to deal with overlapping systems and alert fatigue which is an established barrier [42] particularly relating to patients whose vital signs are typically outside of parameters [32]. Doctors whose engagement was not mandatory may never have been driven to overcome opportunity costs; as seen in other studies, different professional groups can perceive change in different ways [25] and different motivators may be in play [43]. A tipping point exists for change where “evidence of change becomes evidence for change” (43p1); this may not have been reached for the R-VS group.

The ‘perceived effectiveness’ of DETECT e-PEWS was most evident in the reports by nurses (D-VS and R-VS) who valued the system’s effectiveness and clinical utility (e.g. real time reporting, responding quickly, providing accurate data). The system was clearly less effective due to lack of buy-in by doctors; there is insufficient evidence from doctors as to whether the system has clinical utility. All HPs believed that if DETECT e-PEWS was mandatory for everyone, it could be more effective. HPs (R-VS group) were keen to relaunch DETECT e-PEWS. This attitude perhaps acknowledges that implementation is complex and requires sustained effort to overcome barriers [1, 42].

Acceptability in relation to ‘self-efficacy’ was good as DETECT e-PEWS was not considered difficult to use and all HPs were confident in their use, after the initial period of gaining skills. This suggests that clinical utility was robust in this construct.

Acceptability is clearly multifaceted and using the TFA to examine acceptability has shown that a wide lens needs to be adopted to try and understand factors that influence implementation of e-PEWS. Implementation might be better supported by the use of sociotechnical [12] or sociocultural frameworks [44] that take account of the human-interface behavioural aspects of implementing technological change as these are perhaps best placed to support the complexity of change and some of the human barriers encountered in DETECT e-PEWS.

### Limitations

Despite efforts to recruit participants the sample size is small when compared to the number of HPs using the DETECT system. Technology acceptance is a staged process [23] and the interviews reflect that staging; the D-VS group had more experience with DETECT e-PEWS than the R-VS group. Doctors are underrepresented in the sample, reflecting both the COVID-pandemic pressures that existed during recruitment and the lower level of engagement with DETECT e-PEWS. The perspectives of the doctors presented in the paper are not directly reflective of the wider population, and do not present the views of those who resisted/failed to engage with DETECT e-PEWS. However, we did ask those who participated for their perceptions of the opinions of other doctors to try and gain a breadth of understanding. A follow-up evaluation, using the same methods, is planned to be undertaken 12 months after this evaluation.

## Conclusions

This qualitative study demonstrates how initial acceptability can differ across different disciplines. and how the mandate for change can make a difference in how comprehensively technology is embedded in practice. These differences need to be considered when planning future implementations of this nature. Streamlining the number of devices that need to be carried, making the use of the system mandatory for all HPs, and having strong clinical leadership across disciplines that encourages the use of the system are all ways that could facilitate the embedding of DETECT e-PEWS. The DETECT e-PEWS has generated benefits, but these will remain constrained until HPs in both the D-VS and R-VS groups are committed to the system.

## Supplementary Information

**Additional file 1.** Full list of vital signs (and associated steps).

**Additional file 2.** Process for escalation task for ‘deteriorating patient’.

**Additional file 3.** Key elements of managing tasks, reviewing alerts and tagging children (and associated steps).

**Additional file 4.** Interview technique and key steps taken in thematic analysis of data.

**Additional file 5.** Interview schedule.

## Data Availability

The datasets generated and/or analysed during the current study are not publicly available due to limitations within the ethics approval but are available from the corresponding author on reasonable request.
