# Supplementary material for: Health professionals’ initial experiences and perceptions of the acceptability of a whole-hospital, pro-active electronic paediatric early warning system (the DETECT study): a qualitative interview study
Source: BMC Pediatr. 2022 Jun 24;22:365. doi: 10.1186/s12887-022-03411-1 (PMC9233392; doi:10.1186/s12887-022-03411-1)
Supplement: Supplementary file 1 — Additional file 1. Full list of vital signs (and associated steps). [file 12887_2022_3411_MOESM1_ESM.docx]

## Supplementary File 1: Full list of vital signs (and associated steps)

The process of DETECT e-PEWS aims to promote the collection of a complete set of vital signs necessary to be able to determine if the child is deteriorating and to promote situation awareness of potential for child to deteriorate. Vital signs are recorded by registered nurses, healthcare assistants and student nurses.

Prior to starting documentation, the health professional logs in to DETECT e-PEWS and checks or determines whether a new or special assessment is required, confirms patient identification, and checks patient location on the system is correct (e.g., showing the correct ward/unit).

The following set of prompts are presented via the iPod

1. Respiratory rate, clock, unmeasurable, high or extreme values, save.
2. Effort of breathing; normal, abnormal, unmeasurable.
3. Oxygen saturation; unmeasurable.
4. Oxygen requirement; delivery mode, recordings.
5. Heart rate; regular, irregular, unmeasurable.
6. Blood pressure; select limb, lying and standing, unmeasurable.
7. Capillary refill time.
8. AVPU; altered neurology prompts.
9. Temperature.
10. Clinician concern; not concerned.
11. Parent concern; unmeasurable.
12. Summary of observations: check accuracy, correct mistakes, save.
13. Automated categorisation of deterioration risk, clinical prompt acknowledgement.
14. Do you want to contact the clinician?
15. Any suspicion of sepsis? Pre-existing risk displayed or completed here.
16. Does the patient have signs/symptoms of infection?
17. Could this be sepsis? Escalation advice.
18. GCS eye opening, motor, verbal, pupils, power.
19. Blood glucose, pre/post food, blood ketones, insulin.

At the end of entering this information, the next assessment is automatically set by the child’s PEW score. A PEW score of 6 or more will automatically generate a high PEW score (6-9) or Critical PEW score (10+) and will send an alert to the Nurse in Charge and the Clinical Team.
