# Supplementary material for: Health professionals’ initial experiences and perceptions of the acceptability of a whole-hospital, pro-active electronic paediatric early warning system (the DETECT study): a qualitative interview study
Source: BMC Pediatr. 2022 Jun 24;22:365. doi: 10.1186/s12887-022-03411-1 (PMC9233392; doi:10.1186/s12887-022-03411-1)
Supplement: Supplementary file 2 — Additional file 2. Process for escalation task for ‘deteriorating patient’. [file 12887_2022_3411_MOESM2_ESM.docx]

## Supplementary File 2: Process for escalation task for ‘deteriorating patient’

1. Can escalate concerns with/without high PEWS
2. Select required patient from ward list
3. Summary page, contact clinician
4. Email address and password, and/or 5-digit PIN
5. Allow push notifications
6. Escalate Deteriorating patient
7. Allocate to Team (nurse in charge ward specific)
8. Allocate to a person (optional)
9. Urgency
10. Save
11. Track task status by logging into CareFlow Connect part of DETECT e-PEWS on iPod, select task on bottom bar, highlight ‘Tasks I raised’
12. Select the relevant task to view all updates
13. All notes regarding the management and escalation of the deteriorating patient must still be documented in Meditech notes. Timeline from DETECT e-PEWS can be used as an aide memoire.
