# Supplementary material for: Health professionals’ initial experiences and perceptions of the acceptability of a whole-hospital, pro-active electronic paediatric early warning system (the DETECT study): a qualitative interview study
Source: BMC Pediatr. 2022 Jun 24;22:365. doi: 10.1186/s12887-022-03411-1 (PMC9233392; doi:10.1186/s12887-022-03411-1)
Supplement: Supplementary file 3 — Additional file 3. Key elements of managing tasks, reviewing alerts and tagging children (and associated steps). [file 12887_2022_3411_MOESM3_ESM.docx]

## Supplementary File 3: Key elements of managing tasks, reviewing alerts and tagging children (and associated steps)

The process of DETECT e-PEWS aims to promote the collection of a complete set of vital signs that can trigger an alert if a child is showing early signs of serious deterioration or sepsis. Monitoring PEW scores and responding to tasks and alerts raised is undertaken by the nurse in charge, advanced nurse practitioners and by doctors. The processes below are taken from the training plan (a slightly different plan was written for the nurse in charge).

### Managing tasks directed to clinical team re deteriorating child

1. Device will beep when a task is raised
2. Log-into DETECT e-PEWS with 5-digit PIN
3. View task detail
4. Accept task or reassign task, if unable to respond immediately
5. Review patient, agree ongoing management plan, investigations, let me know if [x,y,z] occurs. If reviewing clinician is less than ST3 use team update to make the senior members of the team aware of concerning patients. Complete task. Document the patient review and plan in Meditech, as per current practice.
6. If ongoing clinical concern, despite 1^st^ line management, re-open the previous deteriorating patient task, and reassign to the senior member of the team, with a brief update re the ongoing concern, set the urgency. This means the ward team and the clinical team will see the context of deterioration.
7. Task status can be tracked from the patient list on DETECT e-PEWS

### Viewing patient alerts

1. Device will beep when alert is raised
2. Log-into DETECT e-PEWS with 5-digit PIN
3. View task detail.

### Tagging patients with increased risk for deterioration

1. Log into DETECT e-PEWS, select team patient list
2. Select patient, select 3 dots on the right, select manage tags, select the + sign to the right of the required tags, save
3. Untagging is the same process. Select patient, select 3 dots on the right, select manage tags, remove active tags by tapping the red minus sign to the right of the tag. Select the reason for removal, save.
