# Supplementary material for: Health professionals’ initial experiences and perceptions of the acceptability of a whole-hospital, pro-active electronic paediatric early warning system (the DETECT study): a qualitative interview study
Source: BMC Pediatr. 2022 Jun 24;22:365. doi: 10.1186/s12887-022-03411-1 (PMC9233392; doi:10.1186/s12887-022-03411-1)
Supplement: Supplementary file 4 — Additional file 4. Interview technique and key steps taken in thematic analysis of data. [file 12887_2022_3411_MOESM4_ESM.docx]

## Supplementary File 4: Interview technique and key steps taken in thematic analysis of data

### Interview technique

The interviews were undertaken by HS. HS was a senior Research Fellow (RF) employed on the study. Personal characteristics: PhD, female, social science background, expertise in qualitative health research. Unknown to the participants. In initial conversation about potential participation in study, HS explained she was not a HP, she was employed by Edge Hill university as the RF but was familiar with the hospital as it had been the setting for her PhD.

The interviews were undertaken remotely (during lockdown) by HS who was working from home and with the participants who were either at work or at home. No-one else was present during the interview apart from HS and the participant. Field notes were made, recording context and other issues of relevance. The interviews were all audio-recorded, with the consent of the participants.

The target for the number of interviews in the protocol (n=15-20) was determined pragmatically (based on funding, time available, and the expectation that some data saturation would be reached). However, the Covid pandemic created challenges for recruitment, and we stopped recruitment at the end of the agreed time window. However, we believed we had gained a reasonable level of data saturation within the participant groups/disciplines we had recruited. We also note that the concept of data saturation is contested ^43^; therefore having recruited 14 participants reflecting a diversity of roles, we felt comfortable with recruitment.

All interviews were transcribed by an approved external transcriber and checked by HS. Transcripts were not returned for member checking although HS have all participants the opportunity to clarify and points at the end of each interview and the opportunity to email clarifications, amendments, or additional points after the interview.

### Thematic analysis

The interviews were analysed by BC (experienced qualitative researcher, professor, PhD, children’s nurse, female) and HS. Note: none of the themes or codes were identified a-priori. Analysis was not supported by software. Participants were not asked to provide feedback on the findings as this was an additional burden for HPs already under considerable time pressure.

Analysis followed the five stages of thematic analysis; familiarisation, generating initial codes, searching for themes, reviewing themes and producing report^26^.

None of the themes or codes were identified a-priori. Analysis was not supported by software. Participants were not asked to provide feedback on the findings as this was an additional burden for HPs already under considerable time pressure.

This involved conscientiously reading the interview transcripts (‘familiarisation’), followed by a process of generating preliminary ideas for codes within and across all the transcripts (‘generating initial codes’, such as ‘ease of use’, ‘burden’, ‘confidence/lack of’, ‘compliance’, ‘spatiality’). At every stage the transcripts were first considered within specific sub-groups (nurses, sisters, advanced nurses, doctors) before being considered across these sub-groups. This led to the development of tentative groupings of ideas (‘searching for themes’). A careful review of the tentative themes was undertaken, and a thematic map was produced to reflect the entire data set (‘reviewing themes’) stage. The initial list of themes included themes such as ‘losing your nerve’, ‘persistence of the paper towel’, and ‘messages into the ether’ which were reworked and subsumed. As confidence in the themes developed, the themes were honed and refined until they appropriately reflected the analysis and provided a coherent framework for explaining the findings in a meaningful way. These themes reflected how HPs complied with, circumvented, or disregarded DETECT e-PEWS. Finally, quotations and extracts were selected to illustrate the analysis and themes; these are presented in the paper (‘producing report’). Shortened quotations are presented in the paper and linked to individual participants (by group and number); more extended quotes are presented in a table to demonstrate greater context and the table is organised by theme and participant group. The findings have been presented to reflect both consensus and diverse cases.
