# Supplementary figures and images for: Health professionals’ initial experiences and perceptions of the acceptability of a whole-hospital, pro-active electronic paediatric early warning system (the DETECT study): a qualitative interview study
Source: BMC Pediatr. 2022 Jun 24;22:365. doi: 10.1186/s12887-022-03411-1 (PMC9233392; doi:10.1186/s12887-022-03411-1)

## Supplementary File 5: Interview schedule


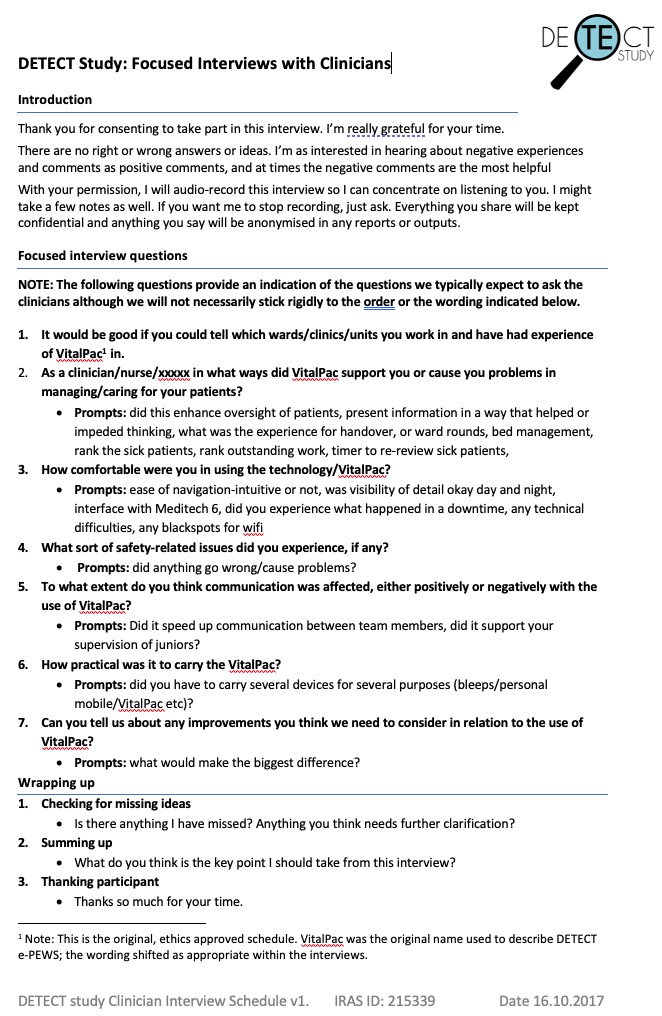

Supplement: Supplementary file 5 — Additional file 5. Interview schedule. [file 12887_2022_3411_MOESM5_ESM.docx]
